# Variable termination sites of DNA polymerases encountering a DNA–protein cross-link

**DOI:** 10.1371/journal.pone.0198480

**Published:** 2018-06-01

**Authors:** Anna V. Yudkina, Antonina P. Dvornikova, Dmitry O. Zharkov

**Affiliations:** 1 Laboratory of Genome and Protein Engineering, Institute of Chemical Biology and Fundamental Medicine, Siberian Branch of the Russian Academy of Sciences, Novosibirsk, Russia; 2 Department of Natural Sciences, Novosibirsk State University, Novosibirsk, Russia; Istituto di Genetica Molecolare, ITALY

## Abstract

DNA-protein cross-links (DPCs) are important DNA lesions induced by endogenous crosslinking agents such as formaldehyde or acetaldehyde, as well as ionizing radiation, cancer chemotherapeutic drugs, and abortive action of some enzymes. Due to their very bulky nature, they are expected to interfere with DNA and RNA synthesis and DNA repair. DPCs are highly genotoxic and the ability of cells to deal with them is relevant for many chemotherapeutic interventions. However, interactions of DNA polymerases with DPCs have been poorly studied due to the lack of a convenient experimental model. We have used NaBH_4_-induced trapping of *E*. *coli* formamidopyrimidine-DNA glycosylase with DNA to construct model DNA polymerase substrates containing a DPC in single-stranded template, or in the template strand of double-stranded DNA, or in the non-template (displaced) strand of double-stranded DNA. Nine DNA polymerases belonging to families A, B, X, and Y were studied with respect to their behavior upon encountering a DPC: Klenow fragment of *E*. *coli* DNA polymerase I, *Thermus aquaticus* DNA polymerase I, *Pyrococcus furiosus* DNA polymerase, *Sulfolobus solfataricus* DNA polymerase IV, human DNA polymerases β, κ and λ, and DNA polymerases from bacteriophages T4 and RB69. Although none were able to fully bypass DPCs in any context, Family B DNA polymerases (T4, RB69) and Family Y DNA polymerase IV were able to elongate the primer up to the site of the cross-link if a DPC was located in single-stranded template or in the displaced strand. In other cases, DNA synthesis stopped 4–5 nucleotides before the site of the cross-link in single-stranded template or in double-stranded DNA if the polymerases could displace the downstream strand. We suggest that termination of DNA polymerases on a DPC is mostly due to the unrelieved conformational strain experienced by the enzyme when pressing against the cross-linked protein molecule.

## Introduction

DNA of all living organisms is perpetually exposed to various exogenous and endogenous genotoxic agents, and is subject to damage [1]. DNA lesions can induce mutations, which lead to cancer and contribute to aging. Since cellular DNA is tightly bound to a variety of structural, regulatory and catalytic proteins, DNA-protein cross-links (DPCs) are among the common types of DNA damage. They can be generated by genotoxic agents such as aldehydes, ionizing and UV radiation, oxidative stress, and some chemotherapy drugs, or through covalent capture of some DNA-dependent enzymes (methyltransferases, topoisomerases) in abortive reaction events [2–4]. Different analytical methods produce estimates of 0.5–70 DPCs per 10^7^ DNA bases as a background level in human cells [5–7].

DPCs as very bulky adducts are expected to interfere with DNA replication and transcription. However, the impact of this type of damage on the genetic machinery remains poorly studied due to the scarcity of suitable experimental models and differences in the emergence mechanisms. The most basic question regarding the genotoxic effects of DPCs is how DNA polymerases behave when encountering these adducts. In a few available model *in vitro* systems DPCs are completely blocking [8–10]. However, at least some DNA polymerases can bypass cross-links with peptides up to 12-amino acids long in an error-free manner [10–13] or with skipping of one or two nucleotides [13].

Known DNA polymerases all share a similar structural fold, consisting of three domains: palm, thumb, and fingers. Based on their sequence and structure similarity and the presence of additional domains, they are currently divided into seven families (A, B, C, D, X, Y and RT) [14–16]. Family A polymerases, best represented by *E*. *coli* DNA polymerase I, are bacterial enzymes responsible for DNA repair synthesis and replacement of Okazaki fragment-initiating primers during replication. Some replicative phage and eukaryotic mitochondrial polymerases also belong here (T7 DNA polymerase, DNA polymerase γ). Family B mostly consists of replicative archaeal, viral and eukaryotic polymerases (DNA polymerases α, δ, and ε, *Pfu* DNA polymerase, DNA polymerases from bacteriophages T4 and RB69). Bacterial replicative polymerases, e. g., *E*. *coli* DNA polymerase III, make up Family C, while a subset of archaeal replicative polymerases is known as Family D. Eukaryotic DNA repair polymerases such as DNA polymerases β and λ form Family X. Family Y covers specialized polymerases from all kingdoms of life, responsible for translesion synthesis and DNA damage tolerance; due to their wide active sites, these polymerases can accommodate many non-canonical nucleotides, including bulky ones, and catalyze error-prone dNTP incorporation. Finally, reverse transcriptases, which can use RNA templates, are grouped in their own Family RT.

Biologically, DNA polymerases would directly encounter DPCs during replication only under special circumstances, since DNA helicases that separate the DNA strands in the replication fork would presumably run into the cross-link first and arrest the fork movement. However, if a DNA helicase travels on the undamaged leading strand and can unwind the DPC, the lagging strand synthesis will run into the cross-link [17]. In other biologically relevant situations such as DNA repair of complex lesions (for example, those induced by ionizing radiation) DNA polymerases could also interact with DPCs. In the analytical setting, DPCs may impede amplification of ancient DNA [18] or DNA from formalin-fixed tissues [19] by polymerase chain reaction. Therefore, it is essential to know how various DNA polymerases would behave when colliding with a DPC. Importantly, all previous studies employed a simple primer/template system with a cross-link in the single-stranded template; no data is available on the situation when DNA synthesis is coupled with the downstream strand displacement, with a DPC in either template or displaced strand of the duplex.

In this paper, we use a fully chemically defined model DPC to investigate the events after its encounter with a variety of DNA polymerases. We report that most DNA polymerases stop when coming into contact with the protein part of a DPC, but some, especially those belonging to Family B, can partially displace the cross-linked protein and continue synthesis even to the point of the cross-link.

## Materials and methods

### Oligonucleotides and enzymes

Wild-type Klenow fragment of *E*. *coli* DNA polymerase I (KF) and phage T4 DNA polymerase were purchased from SibEnzyme (Novosibirsk, Russia). *Taq* and *Pfu* DNA polymerases were from Thermo Scientific (Waltham, MA). *Sulfolobus solfataricus* Dpo4 DNA polymerase was from New England Biolabs (Ipswich, MA). *E*. *coli* Fpg, exonuclease-deficient Klenow fragment of *E*. *coli* DNA polymerase I (KF exo^–^), and bacteriophage RB69 DNA polymerase (RB69pol) were overexpressed and purified essentially as described [20–22]. Human DNA polymerases β and λ and human FEN1 protein were kindly provided by Dr. Olga Lavrik (SB RAS ICBFM). Oligonucleotides (S1 Table) were synthesized from commercially available phosphoramidites (Glen Research, Sterling, VA) and purified by reverse-phase HPLC on a PRP-1 C_18_ column (Hamilton, Reno, NV). The oligonucleotides were 5′-labeled using γ[^32^P]-ATP (PerkinElmer, Waltham, MA) and phage T4 polynucleotide kinase (Biosan, Novosibirsk, Russia) according to the manufacturer’s protocol.

### Model cross-link synthesis

**Fig 1:**
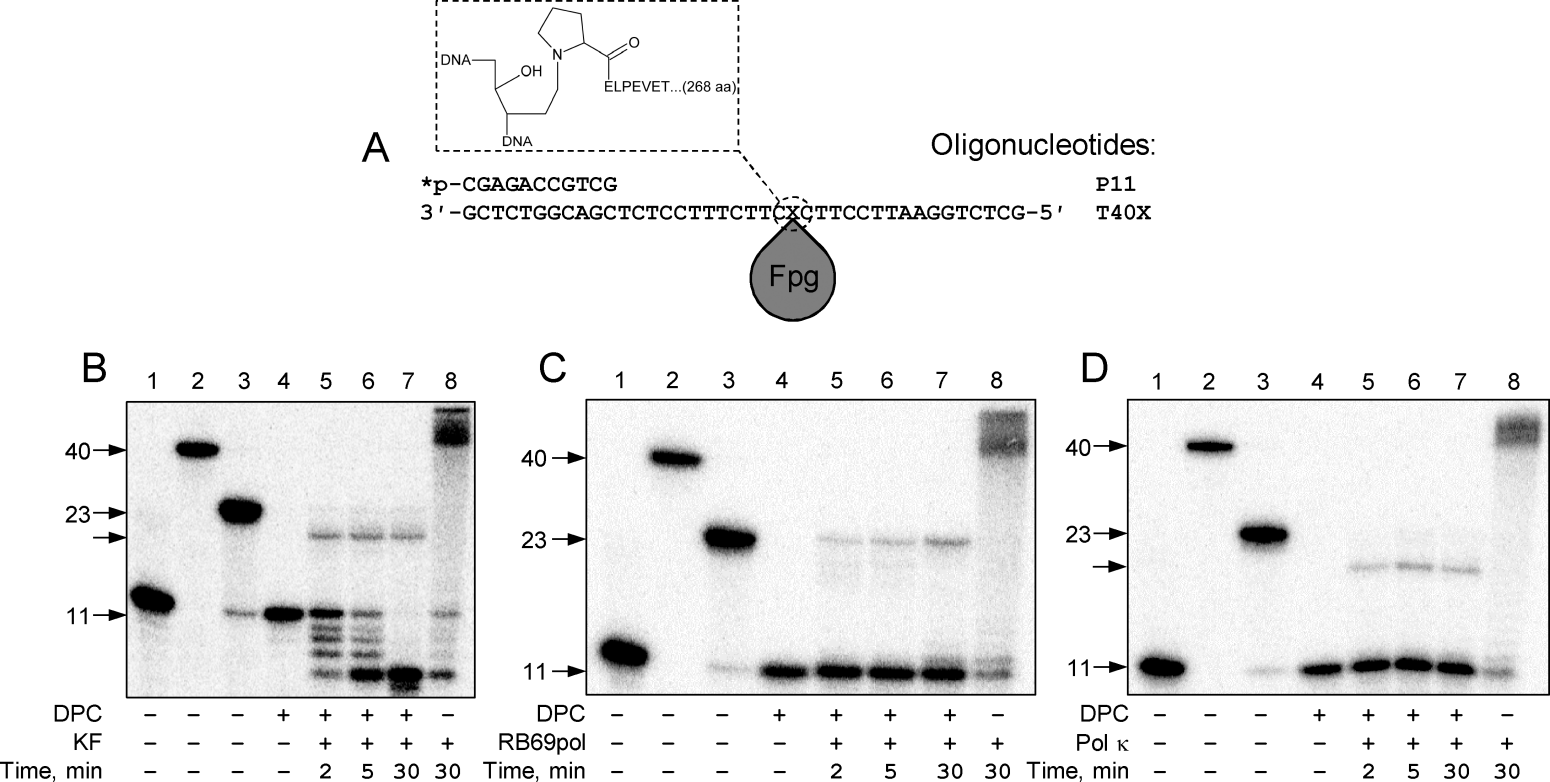
Modes of termination of DNA polymerases at DNA–protein cross-links in the template strand of single-stranded DNA. (A) Scheme of the DPC substrate. The chemical structure of the reduced Schiff base at the site of the cross-link through Pro1 of Fpg is shown in the inset. *p, ^32^P-labeled 5′-end of the primer. (B) Synthesis stops before the cross-link site with the primer degradation: *E*. *coli* DNA polymerase I Klenow fragment (Family A). (C) Synthesis extends to the cross-link site: phage RB69 DNA polymerase (Family B). (D) Synthesis stops before the cross-link site: human DNA polymerase κ (Family Y). *Lanes 1–3*, size markers corresponding to the primer (*lane 1*, 11 nt long), full-size product (*lane 2*, 40 nt), and primer extended to the cross-link site (*lane 3*, 23 nt). *Lane 4*, the substrate in the absence of DNA polymerase, *lanes 5–8*, extension by the DNA polymerase for the indicated time. In *lane 8*, the reaction was carried out with a primer–undamaged template substrate.

**Fig 2:**
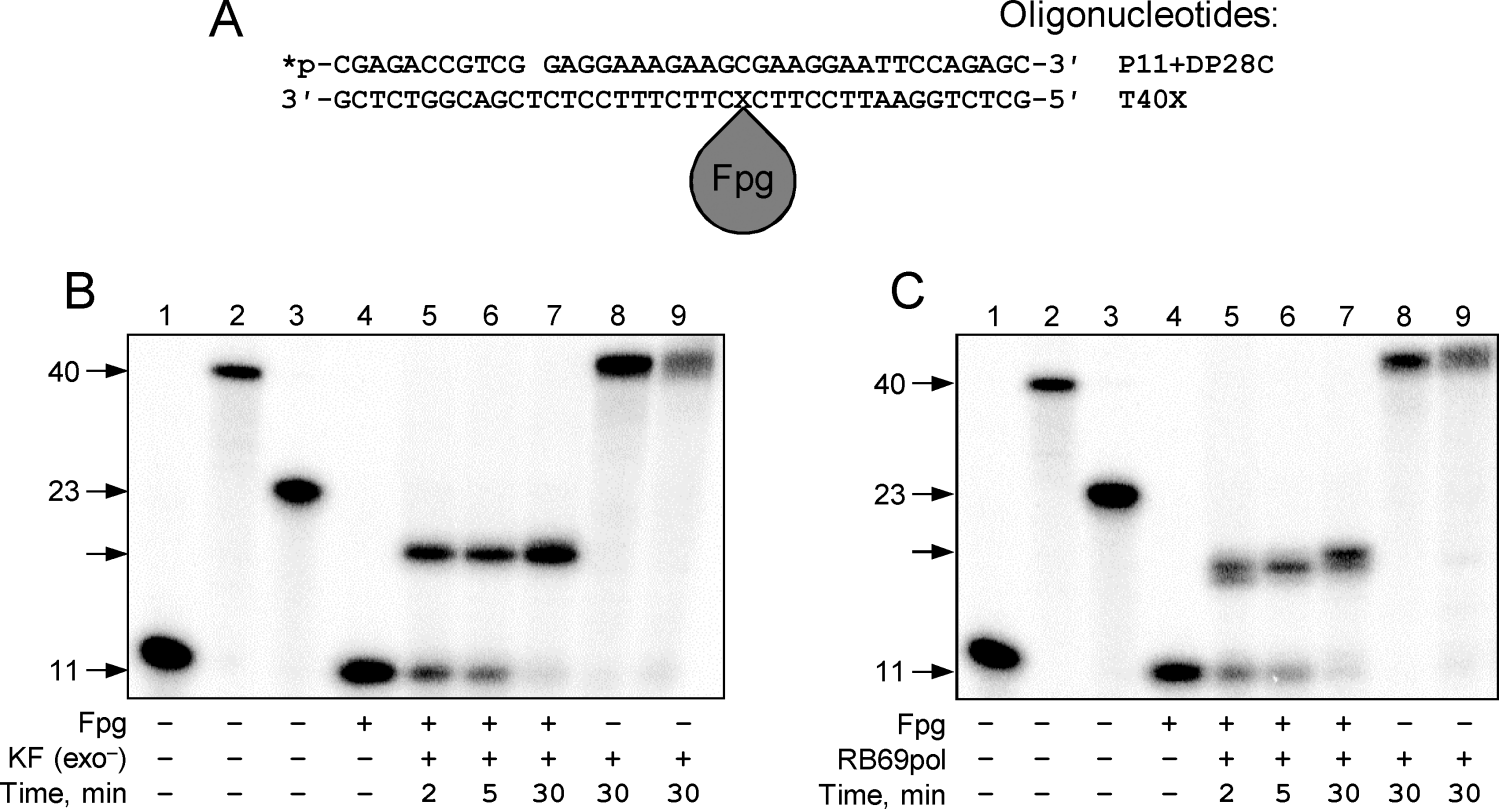
Modes of termination of DNA polymerases at DNA–protein cross-links in the template strand of double-stranded DNA. (A) Scheme of the DPC substrate. (B) Synthesis stops before the cross-link site: KF exo^−^(Family A). (C) Synthesis pauses before and stops closer to the cross-link site: phage RB69 DNA polymerase (Family B). *Lanes 1–3*, size markers corresponding to the primer (*lane 1*, 11 nt long), full-size product (*lane 2*, 40 nt), and primer extended to the cross-link site (*lane 3*, 23 nt). *Lane 4*, the substrate in the absence of DNA polymerase, *lanes 5–9*, extension by the DNA polymerase for the indicated time. In *lane 8*, the reaction was carried out with a primer–undamaged template substrate, and in *lane 9*, with a primer–downstream strand–undamaged template substrate.

**Fig 3:**
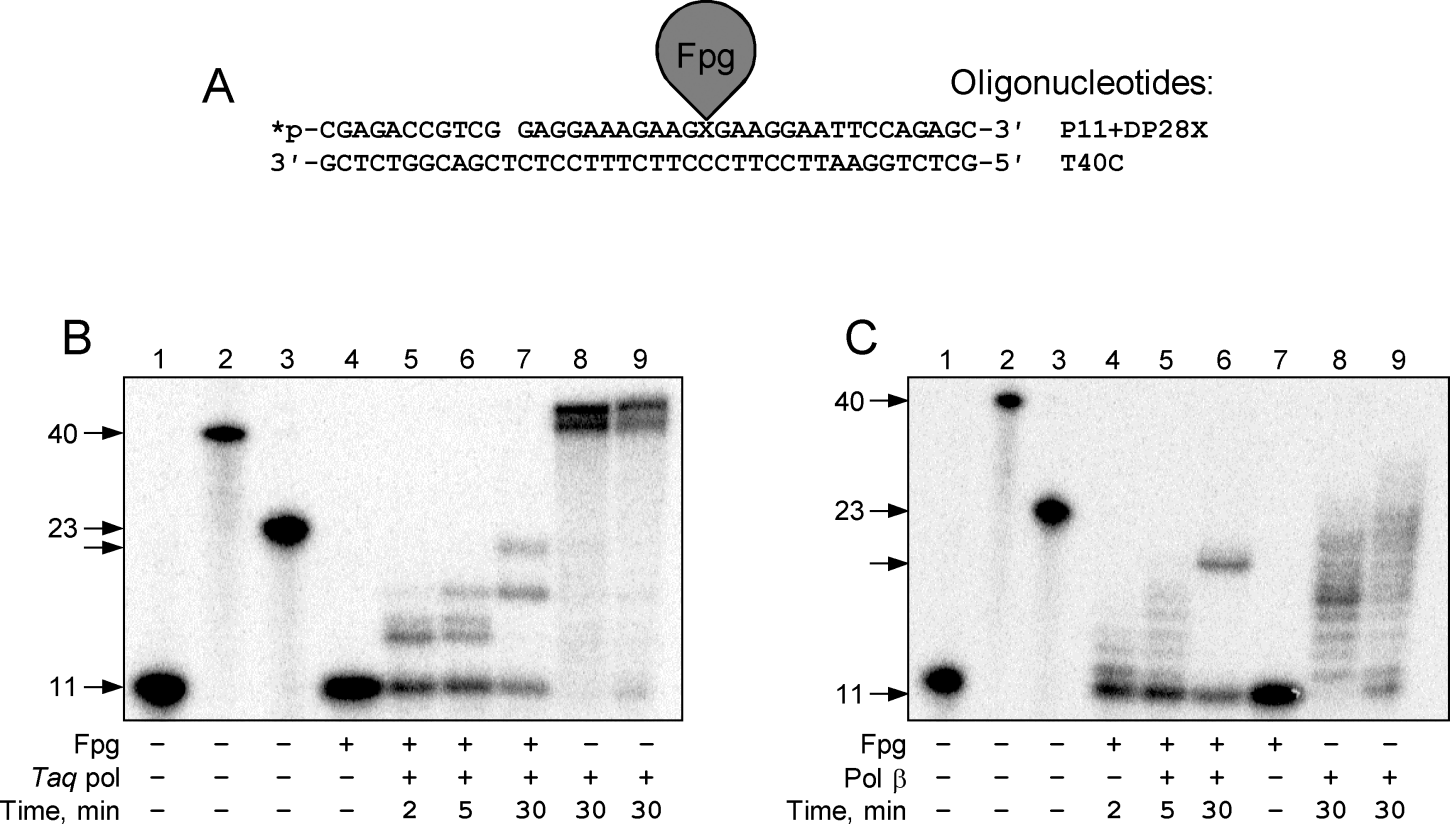
Modes of termination of DNA polymerases at DNA–protein cross-links in the displaced strand of double-stranded DNA. (A) Scheme of the DPC substrate. (B) Synthesis with processive strand displacement with a stop before the cross-link site: *Taq* DNA polymerase (Family A). (C) Synthesis with hit-and-run strand displacement with a stop before the cross-link site: human DNA polymerase β (Family X). *Lanes 1–3*, size markers corresponding to the primer (*lane 1*, 11 nt long), full-size product (*lane 2*, 40 nt), and primer extended to the cross-link site (*lane 3*, 23 nt). *Lane 4*, the substrate in the absence of DNA polymerase, *lanes 5–9*, extension by the DNA polymerase for the indicated time. In *lane 8*, the reaction was carried out with a primer–template substrate, and in *lane 9*, with a primer–undamaged downstream strand–template substrate.

Oligonucleotide duplexes were assembled as shown in Figs 1–3 but without the ^32^P-labeled primer. Two model non-labeled substrates were obtained, one containing oxoG in the template strand, another, in the displaced strand. The reaction mixture contained 50 mM sodium phosphate (pH 6.8), 1 mM ethylenediaminetetraacetate (EDTA), 1 mM dithiothreitol, and 30 pmol of oligonucleotide duplex. A 20-fold molar excess of Fpg and 100 mM NaBH_4_ were added to the reaction mixture simultaneously. The reaction was allowed to proceed at 37°C for 30 min and stopped by adding glucose to 400 mM and incubating for 30 min on ice. The efficiency of cross-linking was 65–95%, as determined by SDS-PAGE. To make a cross-link to the single-stranded template, the reaction was carried out for 60 min under the same conditions using the template strand only. The cross-linking efficiency in this case was typically ~25%. The DNA–Fpg conjugate was purified by 8% non-denaturing PAGE, the band of interest was excised and eluted with 0.1×TE buffer overnight, and the eluate was filtered using the Ultrafree-CL device with 0.22 μm pore diameter (Merck Millipore, Billerica, MA). Purified DPC-containing DNA was concentrated on a Centricon-10 ultrafiltration unit (Merck Millipore). The ^32^P-labeled primer (P11, Table 1) was added and incubated for 1 h at 37°C to complete the assembly of the complex. As controls, we have used primer–template or primer–downstream strand–template constructs without the damaged base, not subjected to the cross-linking procedure.

**Table 1:** Termination sites of DNA polymerases on DPCs.

Family	Poly-merase	DPC in template, single-stranded	DPC in template, double-stranded	DPC in displaced strand
А	KF	4 nt before DPC	5 nt before DPC	3–4 nt before DPC
А	*Taq*	3–4 nt before DPC	5 nt before DPC	3–5 nt before DPC
В	RB69	immediately before DPC	5–6 nt before DPC	immediately before DPC
В	T4	immediately before DPC	no strand displacement, 3′→5′ degradation	no strand displacement, 3′→5′ degradation
В	*Pfu*	4 nt before DPC	no strand displacement^*a*^	no strand displacement
Х	Pol β	4–5 nt before DPC	3–4 nt before DPC	4–5 nt before DPC
Х	Pol λ	3 nt before DPC	8 nt before DPC	8 nt before DPC
Y	Dpo4	immediately before DPC	poor strand displacement^*b*^	poor strand displacement
Y	Pol κ	4 nt before DPC	poor strand displacement	poor strand displacement^*c*^

^*a*^No strand displacement: insertion of a single nucleotide into the gap without further DNA synthesis in the undamaged control substrate.

^*b*^Poor strand displacement: insertion of 3–4 nucleotides without further DNA synthesis in the undamaged control substrate.

^*c*^For Pol κ, very limited progress to 2–3 nt before DPC was observed.

### Primer extension reactions

The reaction mixture (20 μl) contained 500 μM dNTP mixture (with equimolar ratio of all four dNTP), 400 fmol of a DPC substrate (see above) and the required DNA polymerase in the respective optimal buffer: 50 mM Tris–HCl (pH 7.5), 5 mM MgCl_2_, 0.1 mM EDTA, 30 mM KCl; 0.1 mM DTT for KF and KF exo^–^; 20 mM Tris–HCl (pH 8.8), 10 mM (NH_4_)_2_SO_4_, 10 mM KCl, 2 mM MgSO_4_, 0.1% Triton X-100 for *Pfu*, 10 mM Tris–HCl (pH 8.8), 50 mM KCl, 1.5 mM MgCl_2_, 0.08% Nonidet-P40 for *Taq*; 10 mM Tris–HCl (pH 7.6), 10 mM MgCl_2_, 1 mM DTT for Pol β; 50 mM Tris–HCl (pH 8.0), 0.5 mM MgCl_2_, 0.5 mM DTT for Pol λ; 50 mM Tris–HCl (pH 7.5), 5 mM MgCl_2_, 0.1 mM DTT for RB69pol; 67 mM Tris–HCl (pH 8.8), 6.7 mM MgCl_2_, 16.7 mM (NH_4_)_2_SO_4_, 1 mM DTT for T4pol; 50 mM HEPES (pH 7.5), 5 mM MgCl_2_, 60 mM KCl, 5 mM DTT for Pol κ; 20 mM Tris–HCl (pH 8.8), 10 mM (NH_4_)_2_SO_4_, 10 mM KCl, 2 mM MgSO_4_, 0.1% Triton X-100 for Dpo4. The buffers were selected based on the manufacturers’ instructions and literature data. FEN1 was added to 400 nM, if necessary. The quantity of the DNA polymerase was optimized to give ~70% insertion of the first nucleotide in 2 min. The reaction was allowed to proceed at 37°C for 2, 5, or 30 min. At these times, aliquots were withdrawn, an equal volume of formamide dye solution (80% formamide, 20 mM Na-EDTA, 0.1% xylene cyanol, 0.1% bromophenol blue) was added followed by 2-min heating at 95°C, and the reaction products were resolved by 20% denaturing PAGE and visualized by posphorimaging (Typhoon FLA 9500, GE Healthcare, Chicago, IL). The lengths of extension products were determined from comparisons with the mobility markers and partial extension ladders.

## Results

### Model cross-links

To investigate bypass of DPCs by DNA polymerases belonging to different families, we have covalently cross-linked *E*. *coli* formamidopyrimidine-DNA glycosylase (Fpg) to oligonucleotide substrates containing 8-oxoguanine (oxoG) in the presence of NaBH_4_ (Figs 1–3, Panel A). Since DPCs may be formed with a variety of proteins, we have reasoned that there is little specificity in the interaction of DNA polymerases with them, and chose the model based on the easiness of its synthesis. Fpg forms a Schiff base between the enzyme’s active site amino group and C1′ of the damaged nucleoside as a reaction intermediate, which can be reduced to a stable covalent amine [23] (Fig 1A, inset), a reaction that was used to prepare Fpg–DNA conjugate in large quantities for crystallization [20]. Here, Fpg was efficiently cross-linked to double-stranded DNA containing oxoG in either the template strand or the displaced strand (65–95% yield under the conditions described in Materials and Methods). Single-stranded DNA is a much worse substrate for Fpg due to both lower affinity and lower catalytic constant; however, at a large enzyme excess, the reaction can be pushed to a reasonable depth [24]. In our experiments, we obtained a ~25% yield of the cross-link with oxoG in the single-stranded template. The cross-links were purified by non-denaturing gel electrophoresis and used in the bypass studies. The control substrates were assembled with the non-damaged downstream primer DP28C and template T40G (S1 Table).

### Primer extension with DPCs in the single-stranded template by DNA polymerases of various families

Family A DNA polymerases are involved in replication of some bacteriophages (e. g., phage T7 DNA polymerase), replication of mitochondrial DNA in eukaryotes (DNA polymerase γ), and in DNA repair and processing of Okazaki fragments in bacteria (DNA polymerase I) [25]. Interestingly, the Klenow fragment of DNA polymerase I (KF) have been reported to bypass dodecapeptide adducts to *N*^6^ of adenine in an error-free manner [26]. Generally, DNA synthesis on DPC-containing primer–template substrates by family A DNA polymerases can be illustrated by the experiment with KF, which efficiently extended the primer but stopped ~4 nucleotides before the cross-link site (Fig 1B, Table 1). Bands corresponding to products of hydrolysis of the 11-mer primer were observed together with the elongation product due to the 3′→5′ exonuclease activity of KF (Fig 1B). Exonuclease-deficient KF (KF exo^–^) behaved in the same manner but expectedly showed no primer degradation (S2 Fig). Another member of Family A, *Taq* DNA polymerase, stopped before the cross-link at approximately the same position as KF did (S3 Fig, Table 1) and showed several pause points at earlier reaction times.

Family B comprises mainly replicative DNA polymerases having high fidelity and involved in the synthesis of both leading and lagging strands during replication (eukaryotic DNA polymerases α, δ, ε, ζ, some archaeal and phage DNA polymerases). Many of them have high 3′→5′ exonuclease activity (proofreading activity) to remove erroneously incorporated nucleotides not complementary to the template. We have investigated interaction of Family B enzymes with DPCs using DNA polymerases from bacteriophages RB69 and T4 and archaeal *Pfu* DNA polymerase [27–29]. In contrast to Family A polymerases on substrates containing a DPC in the single-stranded template, RB69pol synthesized a 23-mer product, which means that it was able to reach the cross-link site despite the presence of a bulky protein adduct (Fig 1C). T4 DNA polymerase was also able to reach the cross-link site, although due to its strong exonuclease activity significant primer degradation was observed (S4 Fig). The activity of thermostable *Pfu* DNA polymerase, which has a temperature optimum at 72–74°C, was rather low at 37°C. However, it still can be observed that on a substrate with a DPC-containing single-stranded template, *Pfu* DNA polymerase stopped 4–5 nucleotides before the cross-link site, as do Family A polymerases (S6 Fig). It remains to be seen whether *Pfu* DNA polymerase could read through closer to the cross-link site at its optimal temperature.

Family X includes eukaryotic DNA polymerase β (Pol β) involved in the process of short-patch base excision repair [30,31], as well as DNA polymerases λ (Pol λ) and μ likely participating in non-homologous end joining during double-stranded breaks repair [32,33]. We have investigated the activity of two representative members of Family X, Pol β and Pol λ. Unlike the previously described polymerases, these enzymes perform distributive synthesis on primer–template substrates: they catalyze single nucleotide addition to a primer, release DNA substrate, and then this cycle is repeated; however, both polymerases are processive on substrates containing short gaps, interacting with the downstream strand [34–37]. Both Pol β and Pol λ did not pass further than 3–5 nucleotides before the cross-link site, which makes them similar to Family A polymerases (S7 and S8 Figs). However, the efficiency of the polymerase reaction was clearly lower than in the cases of Families A and B, likely reflecting the preference of Family X enzymes for gapped rather than primer–template substrates. Interestingly, both Pol β and Pol λ synthesized longer products in the presence of a DPC than on an undamaged template (Panel A in S7 Fig, Panel A in S8 Fig, compare length of the products in lanes 7 and 8); we speculate that the cross-link in a single-stranded template might provide some non-specific protein–protein interactions downstream that help the polymerase to hold on the template and approach closer to the damage site.

Family Y DNA polymerases stand out in their ability to perform translesion synthesis, that is, to incorporate dNTP opposite damaged DNA bases [38]. This capacity is explained by a short fingers domain and a wide active site, which together allow the polymerase to bind DNA even if it is distorted by damage. The reverse of this flexibility is high error frequency, which is greater by 1–2 orders of magnitude in Family Y DNA polymerases than in other families. Family Y includes DNA polymerases such as human DNA polymerases η, ι, κ (Pol κ), Rev1, and bacterial and archeal DNA polymerases IV and V. In our research we have investigated the activity of Family Y enzymes on substrates containing a DPC using DNA polymerase IV from *Sulfolobus solfataricus* (Dpo4) and human Pol κ. In the experiments with Dpo4 when a DPC was in single-stranded template, we observed several pause points after adding 2, 4 and 8 nucleotides to the primer, which did not occur with the control primer–template substrate (S9 Fig). Interestingly, they all correspond to dGMP incorporation in the growing strand, and the pause may be related to the fact that Dpo4 uses dGTP with the worst kinetics of all possible dNTPs [39]. It is possible that similar kinetic considerations explain the presence of pause points in the control reactions for some other reactions studied polymerases (for instance, *Taq* DNA polymerase). A band of low intensity corresponding to the length of 23 nucleotides was observed after prolonged incubation of Dpo4 with the substrate, which means that this polymerase is able to continue synthesis to the site of the cross-link with some efficiency. Pol κ during synthesis on the same substrate has shown no pause points and stopped 4 nucleotides before the site of the cross-link (Fig 1D).

### Primer extension with DPCs in the template strand of double-stranded DNA

DNA polymerases vary widely in their ability to synthesize the new strand of DNA when the downstream strand is present. Family A DNA polymerases degrade the downstream strand through the activity located in their 5′→3′-exonuclease domain (which is not a true exonuclease but a structure-specific endonuclease, cleaving off short flaps of displaced DNA) [40]. If this domain is removed, their remaining catalytic domain maintains the ability to displace the downstream strand. Family B polymerases range from non-displacing (*Pfu* and T4 polymerases) to strongly displacing (phage RB69 and φ29 polymerases) [41–43]. Family X enzymes weakly to moderately displace the downstream strand through a “hit-and-run” mechanism, adding and displacing nucleotides one by one [44]. Family Y polymerases also show variable strand displacement activity [45,46].

The presence of the second strand in the DPC-containing part of the substrate profoundly affected the ability of all studied DNA polymerases to progress to the cross-link site. T4 and *Pfu* DNA polymerases, in line with their lack of displacement activity on non-damaged templates, added a single nucleotide to fill the gap (Fig 2A) and did not proceed further (S4 and S6 Figs). T4 polymerase also strongly degraded the primer. Dpo4 and Pol κ poorly displaced the downstream strand even from unmodified control duplexes and showed equally low or worse displacement if DPCs were present (S9 and S10 Figs).

Other DNA polymerases showed some extension but stopped earlier than in the absence of the downstream strand. This difference was especially pronounced in RB69pol (5–6 nt before DPC compared with immediately before DPC in the single-stranded template) and Pol λ (8 nt vs 3 nt before DPC in the single-stranded template) (Fig 2C, S8 Fig). The only exception was Pol β that seemed to stop even closer to the DPC (3–4 nt vs 4–5 nt before DPC in the single-stranded template; S7 Fig), which may be related to the general preference of this polymerase for substrates with a downstream strand. Otherwise, Family A DNA polymerases were the least affected by the presence of the downstream strand, inserting only 1–2 nucleotides less than in the situation with DPC in the single-stranded template (Fig 2B, Table 1).

### Primer extension with DPCs in the displaced strand of double-stranded DNA

The situation with DPCs in the displaced strand is especially interesting, since no translesion synthesis is expected and, provided the polymerase could separate the strands held together by the protein cross-linked to one of them, bypass is possible. However, as our results indicate, displacement of the cross-link-bearing strand presents a challenge even for the polymerases with the strongest displacing activity.

Family A DNA polymerases, which are able to displace or degrade the downstream strand, were about as proficient in synthesizing DNA on substrates with a DPC in the downstream strand as when the DPC was located in the single template strand. KF exo^+^ was able to elongate the primer by 8–9 nucleotides, then stopped 3–4 nucleotides before the cross-link site, whereas KF exo^−^pushed even further (S1 and S2 Figs). Another member of Family A, *Taq* DNA polymerase, stopped before the cross-link at about the same position as KF and couldn’t continue synthesis (Fig 3B). Unlike KF, *Taq* polymerase paused in the beginning of elongation after single-nucleotide gap filling but this pause was later overcome by the enzyme. The same pause point was present in the control reaction, indicating that it could be due to suboptimal temperature conditions for strand degradation by this DNA polymerase. Thus, despite Family A DNA polymerases have an ability to tolerate some bulky peptide adducts [26], they were not able to bypass DPCs in any context.

Family B RB69pol was able to progress until very near to the cross-link site, as in the case with the adduct in the single-stranded template (S5 Fig). Asymmetric orientation of Fpg on DNA, as seen in the crystal structure [20], shifts the edge of Fpg footprint by 2 nt in the 3′ direction when the cross-link is in the displaced strand relative to the DPC in the template strand (S11 Fig). However, this difference alone cannot explain the ~7 nt difference in the polymerase termination point, indicating that RB69pol can partially displace the downstream strand even having such a bulky adduct as the DPC. We have inquired whether this displacement can be further assisted by degradation of the downstream DNA by structure-specific endonuclease FEN1. However, no progress of RB69pol beyond the cross-link position was seen even in the presence of FEN1. T4 and *Pfu* DNA polymerases had no strand displacement activity on this type of substrate and behaved identically to their reaction with DPCs in the template in double-stranded DNA (S4 and S6 Figs).

Family X polymerases performed similarly to their activity on substrates with DPCs in the template. Pol β stopped 4–5 nucleotides before the cross-link site whereas Pol λ, with its poorer strand displacement ability, terminated ~8 nucleotides before the cross-link (Fig 3B, S6 Fig). Here again the difference between the termination positions of Pol β when the DPC was in the template and in the displaced strand could not be explained by 2-nt asymmetry in the protein footprint (S11 Fig).

Finally, Family Y DNA polymerases (Dpo4 and Pol κ) showed poor strand displacement with the control substrates. Only Dpo4 at long reaction times showed a weak signal corresponding to extension to the position 2–3 nt before DPC (S9 Fig).

## Discussion

Relative to a typical size of DNA lesions, DNA–protein cross-links are very bulky adducts. As a DPC model, we have used Fpg protein trapped on DNA by NaBH_4_ treatment; according to its crystal structure, Fpg fully or partially covers ~7 nt on the damaged DNA strand, and ~9 nt on the complementary strand [20] (S1 Fig). In our substrates, 8-oxoguanine, the lesion that Fpg excises and gets trapped to this position, was 13 nucleotides from the 3′-terminus of the primer. In turn, DNA polymerases, even though their size varies, are also large molecules (38–114 kDa for polymerases used in our study; cf. 30 kDa for Fpg). Thus, from the steric considerations, DNA polymerase should run into the protein part of the DPC soon after the start of synthesis. The available X-ray structures [47,48] allow us to estimate the distance at the start between the polymerase and Fpg for Pol β and RB69pol. When Fpg is cross-linked to the displaced strand, the surfaces of Pol β and RB69pol are, respectively, 6 nt and 5 nt away from the surface of Fpg. Thus, the termination of Pol β 4–5 nt before the site of the cross-link, correspond to an overlap of 2–3 nt between its footprint and the footprint of Fpg. When Fpg is cross-linked to the template strand, the surfaces of Pol β and Fpg are separated by 4 nt, due to the asymmetry of the Fpg footprint, and the overlap at the termination site is 0–1 nt. Even more strikingly, stalled RB69pol overlaps over at least 4 nt with Fpg in the template strand and nearly with the full footprint of Fpg cross-linked to the displaced strand.

**Fig 4:**
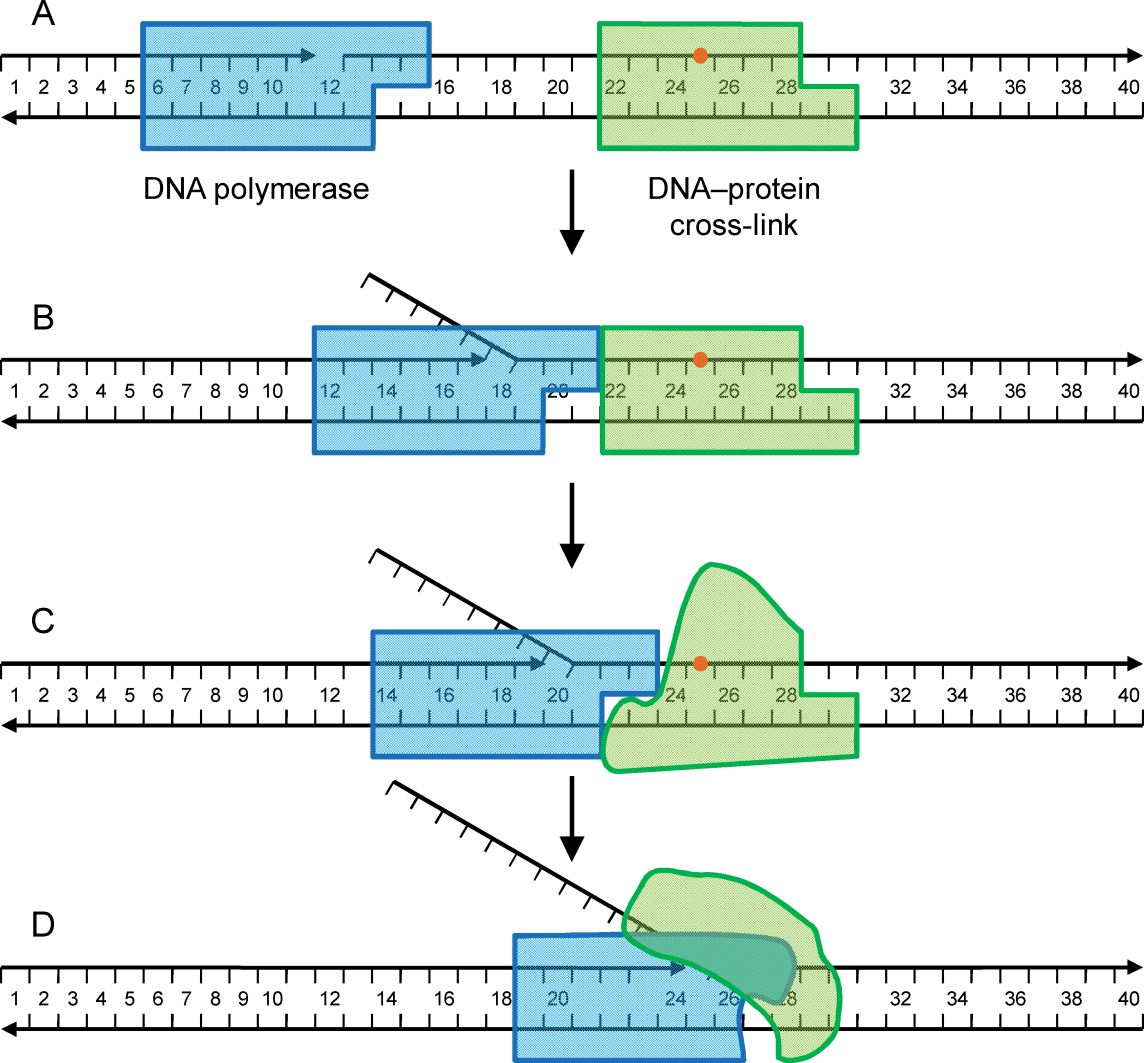
Scheme of the “kiss-and-push” mechanism, shown for substrates containing a DPC in the displaced strand of double-stranded DNA. A, substrate; B, after insertion of several nucleotides, the DNA polymerase comes to contact with the cross-linked protein (the kiss stage); C, the ability of some DNA polymerases to distort the cross-linked protein aids the incorporation of a few extra nucleotides (the push stage); D, the synthesis stops when the polymerase itself gets distorted.

The results with all DNA polymerases studied in this work are summarized in Table 1. Although there was considerable heterogeneity in the position of termination sites, our observations suggest that DNA polymerase interaction with DPCs may be described by a scheme that we term “kiss-and-push model” (Fig 4). The incorporation of nucleotides is mostly guided by the ability of the DNA polymerase to perform synthesis on a given substrate (e. g., by its strand displacement activity) until the surfaces of DNA polymerase and protein in the DPC are in contact (the “kiss” stage). Most polymerases stop at this point but some, especially Family B polymerases and Dpo4 (in single-stranded DNA), can “push” the cross-linked protein to read through up to the site of cross-linking, until the polymerase is distorted to a degree where it cannot catalyze nucleotide incorporation anymore. Even at the kiss stage, it is likely that the protein globules of the polymerase, or the cross-linked protein, or both are partially deformed, since the termination point is closer to the cross-link site than allowed by the size of both proteins. In this respect, the kiss stage resembles the stalling of reverse transcriptases by RNA-binding proteins in the toeprinting assay used to analyze translation complexes; in this case, cDNA synthesis stops at or very close to the 3′-edge of the ribosome or other protein obstacle [49]. Family B polymerases would have the highest ability to distort the protein obstacle.

None of the studied DNA polymerases was able to bypass DPCs. This is not unexpected when a DPC is in the template strand, since accommodation of adducts of this size in the active site should be beyond the capability even for Family Y polymerases, and the attached protein is likely too large to permit template slippage to skip the cross-link. Also, lack of the base at the site of the cross-link would contribute to stalling the polymerase, although simple abasic sites can be bypassed after a pause by polymerases from Families A, B, X, and Y [50–52]. Moreover, a comparison of the results with DPCs in single- and double-stranded template clearly indicates that the displaced strand impedes the progress of DNA polymerases near the bulky protein adduct.

The inability of strand-displacing DNA polymerases to fully extend the primer when the DPC is in the displaced strand is more intriguing. There are no available X-ray structures of DNA polymerases in the strand displacement complex. In a model of the strand displacement complex of phage φ29 DNA polymerase that belongs to Family B [42,53], the displaced strand forms few contacts with the protein. However DPCs are likely bulky enough to jam the polymerase at some point along the path of the displaced strand, or to distort the DNA duplex to a degree that would not allow the incoming polymerase to draw the non-damaged template strand into the active site.

Overall, it is clear that DPCs indeed present an almost insurmountable problem for bypass by DNA polymerases. This necessitates the repair that involves specific proteases, such as Wss1 in yeast and SPRTN in metazoans, which degrade the protein to a cross-linked peptide of a manageable size and are required for replication in the presence of DPC [54–57]. Biallelic mutations in the *SPRTN* gene cause Ruijs–Aalfs syndrome, a hereditary disease characterized by progeroid features, early cancer development, and hypersensitivity to DNA damage, likely a result of accumulation of unrepaired dead-end topoisomerase 1 complexes [58–60]. It would be interesting to see whether the distortion in the cross-linked protein induced by the collision with a polymerase might expose normally buried peptides and activate the nuclear misfolded protein response [61]. In addition, if replication is concerned, interaction of DNA helicases with DPCs could trigger response that stops the replicative fork and prevents the polymerase from collision with DPCs. Family X polymerases, which are involved in DNA repair, stop at least 4–5 nucleotides before the cross-link site. Therefore, in clustered damage where a DPC is combined with a modified DNA base, abasic site, or single-strand break, the types of lesions repaired with participation of Pol β and Pol λ [62], the complete repair could be difficult. These processes present an interesting topic worthy of further research, and the Fpg cross-link system used in this study provides a convenient model, now characterized at the basic level, that could be used *in vitro* or easily introduced into plasmids for experiments in living cells.

## Supporting information

S1 Fig **Termination sites of *E*. *coli* DNA polymerase I Klenow fragment (Family A)** at the DNA–protein cross-link in the template strand of double-stranded DNA **(A)** and in the displaced strand of double-stranded DNA **(B)**. Lanes 1–3, size markers (primer, 11 nt long; primer extended to the cross-link site, 23 nt; full-size product, 40 nt); the arrows indicate their positions. The presence of DNA polymerase, cross-linked Fpg, and the reaction time are shown under the gel images. Control reactions were carried out with an undamaged substrate lacking (lane 8) or containing (lane 9) the displaced strand.
(PDF)

S2 Fig **Termination sites of *E*. *coli* DNA polymerase I Klenow fragment exo**^**−**^**(Family A)** at the DNA–protein cross-link in the template strand of single-stranded DNA **(A)** and in the displaced strand of double-stranded DNA **(B)**. Lanes 1–3, size markers (primer, 11 nt long; primer extended to the cross-link site, 23 nt; full-size product, 40 nt); the arrows indicate their positions. The presence of DNA polymerase, cross-linked Fpg, and the reaction time are shown under the gel images. Control reactions were carried out with an undamaged substrate lacking (lane 8) or containing (lane 9) the displaced strand.
(PDF)

S3 Fig **Termination sites of *Taq* polymerase (Family A)** at the DNA–protein cross-link in the template strand of single-stranded **(A)** or double-stranded DNA **(B)**. Lanes 1–3, size markers (primer, 11 nt long; primer extended to the cross-link site, 23 nt; full-size product, 40 nt); the arrows indicate their positions. The presence of DNA polymerase, cross-linked Fpg, and the reaction time are shown under the gel images. Control reactions were carried out with an undamaged substrate lacking (lane 8) or containing (lane 9) the displaced strand.
(PDF)

S4 Fig **Termination sites of phage T4 DNA polymerase (Family B)** at the DNA–protein cross-link in the template strand of single-stranded **(A)** or double-stranded DNA **(B)** and in the displaced strand of double-stranded DNA **(C)**. Lanes 1–3, size markers (primer, 11 nt long; primer extended to the cross-link site, 23 nt; full-size product, 40 nt); the arrows indicate their positions. The presence of DNA polymerase, cross-linked Fpg, and the reaction time are shown under the gel images. Control reactions were carried out with an undamaged substrate lacking (lane 3 in panel B; lane 8 in panels A, C) or containing (lane 4 in panel B; lane 9 in panel C) the displaced strand.
(PDF)

S5 Fig Termination sites of phage RB69 DNA polymerase (Family B) at the DNA–protein cross-link in the displaced strand of double-stranded DNA. Lanes 1–3, size markers (primer, 11 nt long; primer extended to the cross-link site, 23 nt; full-size product, 40 nt); the arrows indicate their positions. The presence of DNA polymerase, cross-linked Fpg, and the reaction time are shown under the gel images. Control reactions were carried out with an undamaged substrate lacking (lane 8) or containing (lane 9) the displaced strand.
(PDF)

S6 Fig **Termination sites of *Pfu* DNA polymerase (Family B)** at the DNA–protein cross-link in the template strand of single-stranded **(A)** or double-stranded DNA **(B)** and in the displaced strand of double-stranded DNA **(C)**. Lanes 1–3, size markers (primer, 11 nt long; primer extended to the cross-link site, 23 nt; full-size product, 40 nt); the arrows indicate their positions. The presence of DNA polymerase, cross-linked Fpg, and the reaction time are shown under the gel images. Control reactions were carried out with an undamaged substrate lacking (lane 7 in panel A; lane 8 in panels B, C) or containing (lane 9) the displaced strand.
(PDF)

S7 Fig **Termination sites of human DNA polymerase β (Family X)** at the DNA–protein cross-link in the template strand of single-stranded **(A)** or double-stranded DNA **(B)**. Lanes 1–3, size markers (primer, 11 nt long; primer extended to the cross-link site, 23 nt; full-size product, 40 nt); the arrows indicate their positions. The presence of DNA polymerase, cross-linked Fpg, and the reaction time are shown under the gel images. Control reactions were carried out with an undamaged substrate lacking (lane 7 in panel A, lane 8 in panel B) or containing (lane 9 in panel B) the displaced strand.
(PDF)

S8 Fig **Termination sites of human DNA polymerase λ (Family X)** at the DNA–protein cross-link in the template strand of single-stranded **(A)** or double-stranded DNA **(B)** and in the displaced strand of double-stranded DNA **(C)**. Lanes 1–3, size markers (primer, 11 nt long; primer extended to the cross-link site, 23 nt; full-size product, 40 nt); the arrows indicate their positions. The presence of DNA polymerase, cross-linked Fpg, and the reaction time are shown under the gel images. Control reactions (lane 8) were carried out with an undamaged substrate lacking the displaced strand.
(PDF)

S9 Fig **Termination sites of *S*. *solfataricus* DNA polymerase IV (Family Y)** at the DNA–protein cross-link in the template strand of single-stranded **(A)** or double-stranded DNA **(B)** and in the displaced strand of double-stranded DNA **(C)**. Lanes 1–3, size markers (primer, 11 nt long; primer extended to the cross-link site, 23 nt; full-size product, 40 nt); the arrows indicate their positions. The presence of DNA polymerase, cross-linked Fpg, and the reaction time are shown under the gel images. Control reactions were carried out with an undamaged substrate lacking (lane 7 in panel A, lane 8 in panels B, C) or containing (lane 9 in panels B, C) the displaced strand.
(PDF)

S10 Fig **Termination sites of human DNA polymerase κ (Family Y)** at the DNA–protein cross-link in the template strand **(A)** or in the displaced strand of double-stranded DNA **(B)**. Lanes 1–3, size markers (primer, 11 nt long; primer extended to the cross-link site, 23 nt; full-size product, 40 nt); the arrows indicate their positions. The presence of DNA polymerase, cross-linked Fpg, and the reaction time are shown under the gel images. Control reactions were carried out with an undamaged substrate lacking (lane 8) or containing (lane 9) the displaced strand.
(PDF)

S11 Fig **A,** arrangement of DNA polymerases and the Fpg cross-link on DNA with sizes of the proteins inferred from the structural data. PDB IDs for structures of Fpg, Pol β and RB69 polymerase are indicated. Arrowheads mark the 3′ termini of the oligonucleotides. The orange dot shows the site of cross-linking. The sphere pattern marks the approximate position of the RB69 domain absent from the crystal structure. **B,** termination positions of Pol β at the Fpg cross-link covalently bound to the displaced strand or the template strand. Blue arrows mark the position of the last incorporated dNMP; red arrows, the corresponding position of the front side of Pol β.
(PDF)

S1 Table Oligonucleotides used in this work. (PDF)
