# Supplementary material for: Variable termination sites of DNA polymerases encountering a DNA–protein cross-link
Source: PLoS One. 2018 Jun 1;13(6):e0198480. doi: 10.1371/journal.pone.0198480 (PMC5983568; doi:10.1371/journal.pone.0198480)
Supplement: S1 Fig — Termination sites of E. coli DNA polymerase I Klenow fragment (Family A) at the DNA–protein cross-link in the template strand of double-stranded DNA (A) and in the displaced strand of double-stranded DNA (B). Lanes 1–3, size markers (primer, 11 nt long; primer extended to the cross-link site, 23 nt; full-size product, 40 nt); the arrows indicate their positions. The presence of DNA polymerase, cross-linked Fpg, and the reaction time are shown under the gel images. Control reactions were carried out with an undamaged substrate lacking (lane 8) or containing (lane 9) the displaced strand. (PDF) [file pone.0198480.s001.pdf]

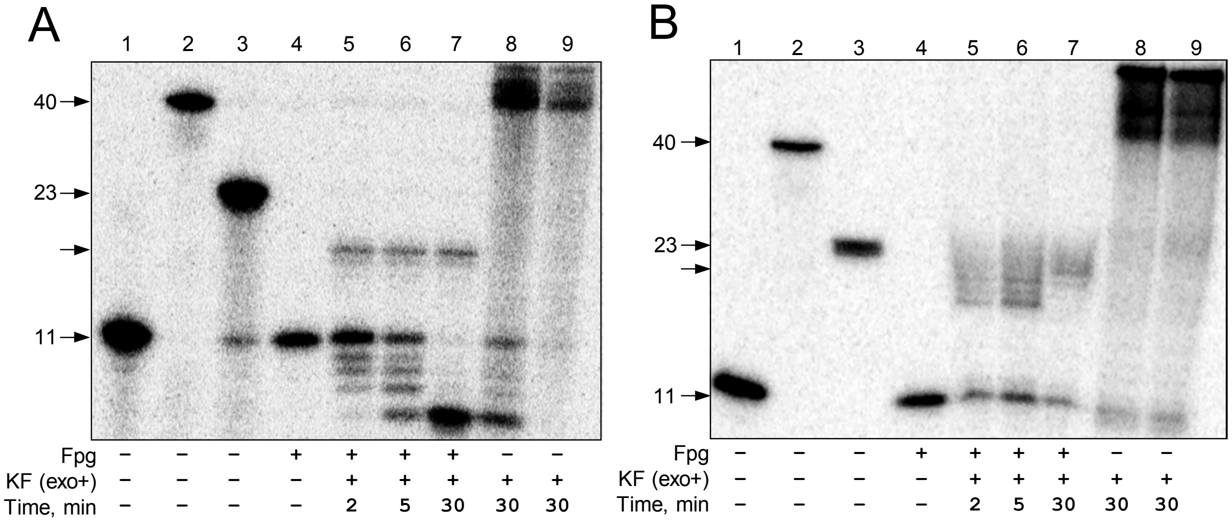

Supplementary Fig. 1.
Termination sites of *E. coli* DNA polymerase I Klenow fragment (Family A) at the DNA–protein cross-link in the template strand of double-stranded DNA (A) and in the displaced strand of double-stranded DNA (B). Lanes 1–3, size markers (primer, 11 nt long; primer extended to the cross-link site, 23 nt; full-size product, 40 nt); the arrows indicate their positions. The presence of DNA polymerase, cross-linked Fpg, and the reaction time are shown under the gel images. Control reactions were carried out with an undamaged substrate lacking (lane 8) or containing (lane 9) the displaced strand.
