# Supplementary material for: Variable termination sites of DNA polymerases encountering a DNA–protein cross-link
Source: PLoS One. 2018 Jun 1;13(6):e0198480. doi: 10.1371/journal.pone.0198480 (PMC5983568; doi:10.1371/journal.pone.0198480)
Supplement: S6 Fig — Termination sites of Pfu DNA polymerase (Family B) at the DNA–protein cross-link in the template strand of single-stranded (A) or double-stranded DNA (B) and in the displaced strand of double-stranded DNA (C). Lanes 1–3, size markers (primer, 11 nt long; primer extended to the cross-link site, 23 nt; full-size product, 40 nt); the arrows indicate their positions. The presence of DNA polymerase, cross-linked Fpg, and the reaction time are shown under the gel images. Control reactions were carried out with an undamaged substrate lacking (lane 7 in panel A; lane 8 in panels B, C) or containing (lane 9) the displaced strand. (PDF) [file pone.0198480.s006.pdf]

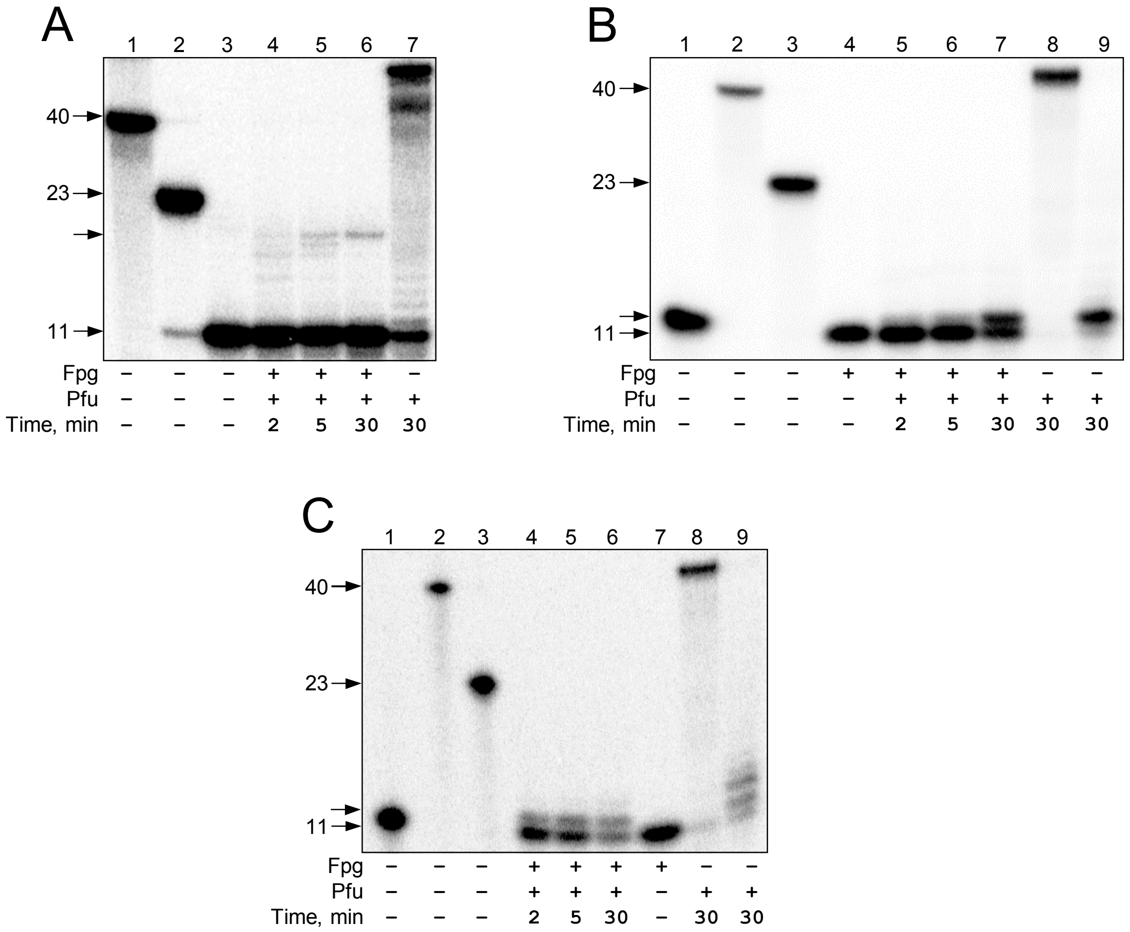

Supplementary Fig. 6.

Termination sites of *Pfu* DNA polymerase (Family B) at the DNA–protein cross-link in the template strand of single-stranded (A) or double-stranded DNA (B) and in the displaced strand of double-stranded DNA (C). Lanes 1–3, size markers (primer, 11 nt long; primer extended to the cross-link site, 23 nt; full-size product, 40 nt); the arrows indicate their positions. The presence of DNA polymerase, cross-linked Fpg, and the reaction time are shown under the gel images. Control reactions were carried out with an undamaged substrate lacking (lane 7 in panel A; lane 8 in panels B, C) or containing (lane 9) the displaced strand.
