# Supplementary material for: Variable termination sites of DNA polymerases encountering a DNA–protein cross-link
Source: PLoS One. 2018 Jun 1;13(6):e0198480. doi: 10.1371/journal.pone.0198480 (PMC5983568; doi:10.1371/journal.pone.0198480)
Supplement: S11 Fig — A, arrangement of DNA polymerases and the Fpg cross-link on DNA with sizes of the proteins inferred from the structural data. PDB IDs for structures of Fpg, Pol β and RB69 polymerase are indicated. Arrowheads mark the 3′ termini of the oligonucleotides. The orange dot shows the site of cross-linking. The sphere pattern marks the approximate position of the RB69 domain absent from the crystal structure. B, termination positions of Pol β at the Fpg cross-link covalently bound to the displaced strand or the template strand. Blue arrows mark the position of the last incorporated dNMP; red arrows, the corresponding position of the front side of Pol β. (PDF) [file pone.0198480.s011.pdf]

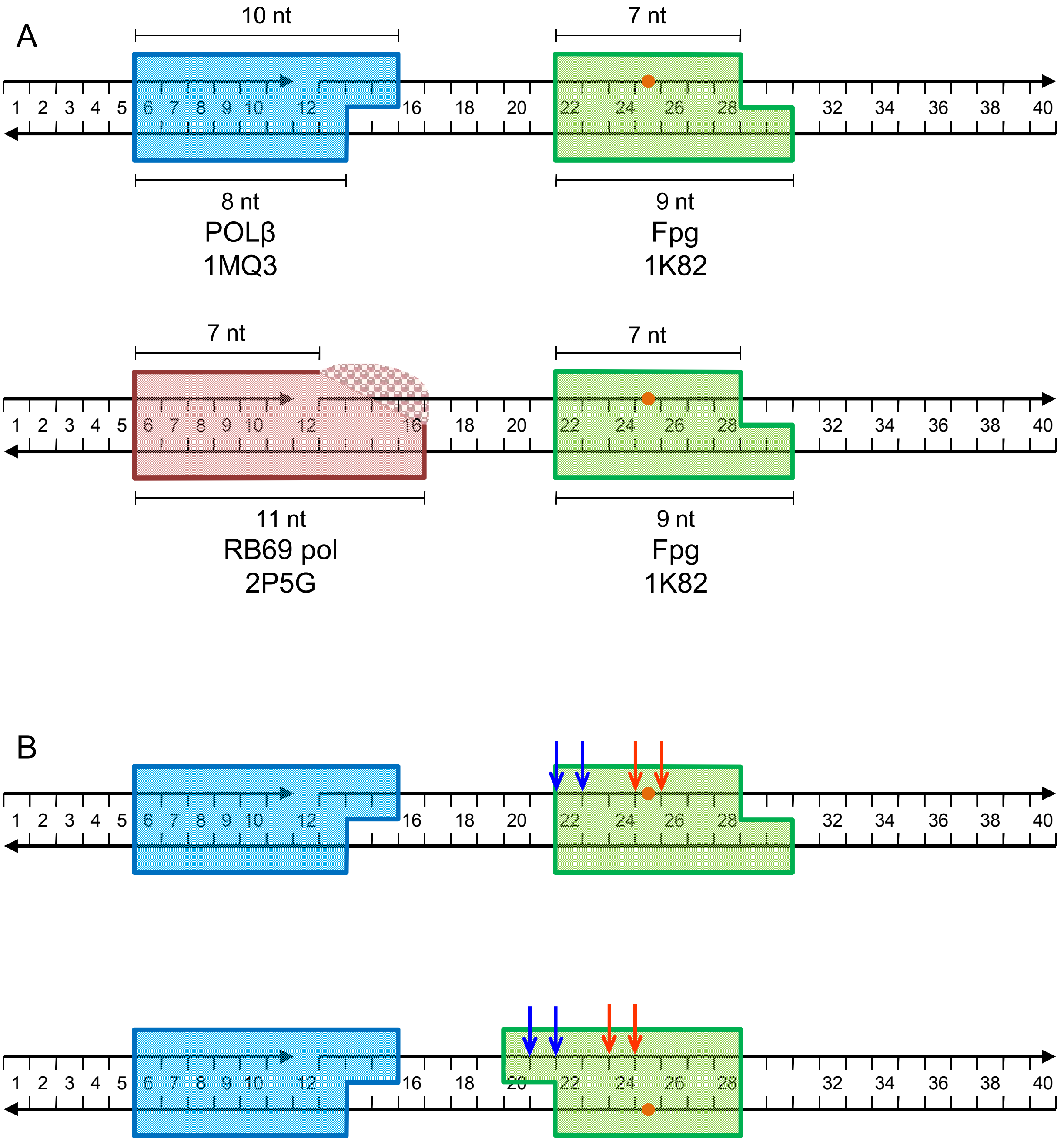

Supplementary Fig. 11.
A, arrangement of DNA polymerases and the Fpg cross-link on DNA with sizes of the proteins inferred from the structural data. PDB IDs for structures of Fpg, Pol β and RB69 polymerase are indicated. Arrowheads mark the 3′ termini of the oligonucleotides. The orange dot shows the site of cross-linking. The sphere pattern marks the approximate position of the RB69 domain absent from the crystal structure. B, termination positions of Pol β at the Fpg cross-link covalently bound to the displaced strand or the template strand. Blue arrows mark the position of the last incorporated dNMP; red arrows, the corresponding position of the front side of Pol β.
