# Supplementary material for: Variable termination sites of DNA polymerases encountering a DNA–protein cross-link
Source: PLoS One. 2018 Jun 1;13(6):e0198480. doi: 10.1371/journal.pone.0198480 (PMC5983568; doi:10.1371/journal.pone.0198480)
Supplement: S1 Table — (PDF) [file pone.0198480.s012.pdf]

S1 Table. Oligonucleotides used in this work

Name	Sequence (5′→3′)[a]
P11	CGAGACCGTCG
M23	CGAGACCGTCGCGAGGAAAGAAG
DP28C	GAGGAAAGAAGCGAAGGAATTCCAGAGC
DP28X	GAGGAAAGAAGXGAAGGAATTCCAGAGC
M40	CGAGACCGTCGCGAGGAAAGAAGCGAAGGAATTCCAGAGC
T40C	GCTCTGGAATTCCTTCCCTTCTTTCCTCTCGACGGTCTCG
T40G	GCTCTGGAATTCCTTCGCTTCTTTCCTCTCGACGGTCTCG
T40X	GCTCTGGAATTCCTTCXCTTCTTTCCTCTCGACGGTCTCG

[a]X, 8-oxoguanine
